# Pediatric Palliative Care in Oncology: Basic Principles

**DOI:** 10.3390/cancers14081972

**Published:** 2022-04-13

**Authors:** Franca Benini, Irene Avagnina, Luca Giacomelli, Simonetta Papa, Anna Mercante, Giorgio Perilongo

**Affiliations:** 1 Paediatric Palliative Care, Pain Service, Department of Women’s and Children’s Health, University of Padua, 35127 Padua, Italy; irene.avagnina@unipd.it; 2 Polistudium Srl, 20135 Milan, Italy; luca.giacomelli@polistudium.it (L.G.); simonetta.papa@polistudium.it (S.P.); 3 Pediatric Neurology and Neurophysiology Unit, Department of Women’s and Children’s Health, University of Padua, 35127 Padua, Italy; anna.mercante@aopd.veneto.it (A.M.); giorgio.perilongo@unipd.it (G.P.)

**Keywords:** pediatric palliative care, PPC, pediatric oncology, pediatric cancer

## Abstract

**Simple Summary:**

About 4 million children with an oncological disease worldwide require palliative care due to the nature of their condition. The WHO defines pediatric palliative care (PPC) as the prevention and relief of suffering in patients with life-threatening or life-limiting disease and their families. PPC relies on the comprehensive and multidisciplinary management of the child and the family’s physical, psychological, spiritual, and social needs. Importantly, PPC begins at the diagnosis of incurability, or supposed incurability, and continues regardless of whether the patient receives any oncological treatment. As such, PPC is a general approach continuing over the entire disease trajectory, which includes, but is not limited to, end-of-life care. This review addresses the value of integrating PPC in treating children with cancer, focusing on the basic principles of PPC and its application in pediatric oncology.

**Abstract:**

About 4 million children with an oncological disease worldwide require pediatric palliative care (PPC) due to the nature of their condition. PPC is not limited to end-of-life care; it is a general approach continuing over the entire disease trajectory, regardless of whether the patient receives any oncological treatment. This review addresses the value of integrating PPC in treating children with cancer, focusing on the basic principles of PPC and its application in pediatric oncology. Moreover, models for PPC implementation in oncology, end-of-life care, and advanced care planning are discussed.

## 1. Introduction

Although major improvements have been achieved in pediatric cancer treatment over the last decades, about 4 million children with oncological diseases worldwide require palliative care due to the nature of their condition [1,2,3]. The WHO defines pediatric palliative care (PPC) as the prevention and relief of suffering in patients with life-threatening or life-limiting disease, as well as for their families [4]. PPC relies on the comprehensive and multidisciplinary management of the child and the family’s physical, psychological, spiritual, and social needs. Importantly, PPC begins at a diagnosis of incurability or supposed incurability, and continues regardless of whether the patient receives any oncological treatment [5]. As such, PPC is a general approach continuing over the entire disease trajectory, which includes, but is not limited to, end-of-life (EoL) care [5,6,7]. Recently, international standards for PPC have been published [5].

It is widely accepted that the implementation of PPC in pediatric oncology improves the quality of life (QoL) of the child and family, helps reduce symptom burden, diminishes costs of care and access to intensive care units at EoL, and makes dying at home more frequent [8,9,10]. Of note, PPC implementation is not associated with shorter survival of the patient [11]. Despite the wide availability of dedicated literature and the numerous advantages of PPC implementation in the management of pediatric patients, PPC is still poorly implemented in oncology practice [12].

Indeed, 56% of children with cancer do not have access to PPC before death, and 44.2% of children with advanced cancer are referred to PPC only at EoL [13,14]. Moreover, only 5% of pediatric oncologists and the child’s family discuss palliative advanced care planning [15]. Some barriers persist and prevent a standardized approach to PPC in settings with limited resources and high-income countries [16,17,18]. These barriers include poor access to essential analgesic drugs in many countries, lack of services and providers dedicated to PPC, scant allocation of resources, limited development of care models, and non-specific educational curricula for healthcare providers [16,19,20,21,22]. Furthermore, PPC is often misperceived as a mutually exclusive service from pediatric oncologists by healthcare providers, patients, and families, and is often associated only with EoL care [8]. Due to all of these barriers, it is not surprising that PPC integration in pediatric oncological practice remains suboptimal. Of note, evidence in the literature suggests that most pediatric oncologists feel that PPC should be consulted more frequently than currently. Thus, further research exploring these specific barriers is necessary to understand the disconnection between oncologists’ attitudes and PPC consultation [18].

This paper discusses the value of integrating PPC in treating children with cancer and discusses the basic principles underlying this integration. More specific guidance on symptom management is beyond the scope of the present article.

## 2. PPC in Oncology: Basic Principles

### 2.1. The Evolving Scenario of Pediatric Oncology

To better understand the basic principles of PPC in oncology, it is of the utmost importance to consider the wide variability in the prognosis and disease trajectory across different types of pediatric cancer.

Indeed, cancer rates in children and adolescents have been steadily increasing since 1975, with leukemia and brain tumors being the most frequently diagnosed [23]. This increase is paralleled by a marked increase in survival rates at 5 years, from 58% in the 1970s to >80% nowadays [24,25]. Prognosis was also varied: in the 1970s, the poorest surviving rates were found in leukemias; nowadays, despite the general improvement in survival, solid tumors, and particularly rhabdomyosarcoma, bone tumors, central nervous system tumors, and neuroblastoma, are associated with the worst prognosis [26]. Moreover, advances in therapeutic approaches have led to a dramatic change in cancer trajectory, which is reflected not only in the above-mentioned improvement in survivorship, but also in a transition of cancer from an acute disease to a chronic condition that includes complex medical needs and medical device dependence [27,28]. The clinical and natural history is different according to the type of cancer. Hematological malignancies in younger children with acute onset are associated with life-threatening symptoms and sudden death (due to disease progression or acute complications), occurring mostly in hospitals. Solid tumors are more common among adolescents, may have a chronic evolution, have a relevant social burden due to the long course of the diseases, and often present with a progressive deterioration before death.

### 2.2. Basic Principles of PPC and Their Application in Pediatric Oncology

Several conditions fall into the domain of PPC applications and can be classified as life-threatening (high probability of premature death, but also a chance of long-term survival), life-limiting (where there is no reasonable hope of cure; both definitions also refer to serious illness), and terminal [5]. PPC is different from palliative care in the adult patient as it has to take into account the particular characteristics of the pediatric patient at a physical, developmental, psychosocial, ethical, and spiritual level [5,29]. Furthermore, parents have a major role in all decisions and retain legal responsibilities regarding their children.

Therefore, patients in PPC and their families should be integrated into specific care programs according to the available resources [21,30,31]. These programs should be implemented at diagnosis or even before diagnosis when this is uncertain [5]. Three levels of PPC delivery can be identified, as follows: (i) palliative approach provided by all healthcare providers; (ii) generalized PPC provided by the oncologist with training in PPC; or (iii) specialized PPC, provided in a dedicated setting by a team of interdisciplinary PPC experts [32,33,34]. The level of PPC delivery can change along the entire disease trajectory.

Care can be provided in various settings, e.g., at home, in hospitals, in ambulatory care, or in a pediatric hospice, according to the clinical severity and complexity of needs. Current evidence emphasizes the importance of ensuring care in the setting of the preference of the child and family, but there is no univocal system applicable to all. A “floating” activity, as described by Brock et al., in which the specialized pediatric palliative care team performs a continuous activity divided between the hospice inpatient, hospitalized inpatient with consultation, and home assistance with 24/7 phone consultation plus domiciliary access could be the response to guarantee the continuum of care, which is unusual for PPC [35,36]. Nevertheless, care at home is generally a common desire of both children and families. Home care ensures a family environment without the additional discomfort of moving to a less familiar and friendly environment, such as a hospital or hospice. Moreover, home care allows for social inclusion in the family’s and friends’ network, counteracting the loneliness that often affects families of seriously ill children [35,37].

A general palliative approach and supportive care are mandatory in all cancer children. Of note, specialized PPC should be provided well before the EoL period in a child with an incurable or potentially incurable oncological disease, considering the prognosis, the physiological changes related to growth, and the complexity of care [12,30]. Indeed, the earlier introduction of PPC support in oncological patients has improved QoL and symptom burden [38]. The early integration of PPC also helps in the development of a trusting relationship between healthcare providers and families, thus improving the decision-making process [5].

To help understand when to seek a specialized level of PPC service, some “green lights” have been proposed (Table 1) [30,31]. In addition to the “green lights”, pediatric oncologists may benefit from using dedicated tools to define the complexity of needs. The pediatric palliative screening scale (PaPas) helps identify children with cancer in need of PPC support by assessing five domains (trajectory of disease, expected outcome and burden from treatments, symptoms burden, preference of care of the child/family, and estimated life expectancy) [39,40]. Its regular use has been associated with an earlier introduction of PPC and subsequent improvement in QoL [41]. Another example is the ACCAPED scale, which defines eligibility to the different levels of PPC delivery by measuring the grade of the complexity of medical needs and the risk of life-threatening events [42]. Nevertheless, using these instruments should not replace the specific evaluation of each case and the development of a trusting relationship. The investigation of the “needs of care” still represents the best approach to understanding the actual needs of the child and family.

## 3. Needs in Pediatric Oncology

Any plan for PPC must provide interventions that balance the risks and benefits by considering both the child’s and family’s QoL, the availability of resources, and local possibilities [5]. On these bases, each plan must address the physical, social, psychological, spiritual, and ethical needs and concerns of patients. Therefore, the assessment of all clinical, communication, psychosocial, and spiritual needs of the patient and family should be performed at the diagnosis of incurability or supposed incurability—or even before if the diagnosis is uncertain—and then periodically during the entire disease course. Furthermore, healthcare providers have their own needs that must be identified and addressed.

An overview of the PPC interventions that should be provided during the disease trajectory and after death is presented in Table 2. The discussion about the specific needs is provided in the following paragraphs.

### 3.1. Child Needs

Clinical needs of children with cancer may vary based on the type of disease, treatment-related possible complications, and previous or acquired comorbidity and change along the trajectory of the disease. Furthermore, the peculiar characteristics of children with cancer should be considered while assessing their needs.

The treatment of symptoms in pediatric oncology must be integrated into the child’s comprehensive care without affecting their cancer-related treatment. The most frequent physical symptoms in children with advanced cancer are chronic pain and fatigue, followed by respiratory problems, nausea and vomiting, cachexia and nutrition intolerance, and constipation [43,44]. In PPC, a multimodal pharmacologic and nonpharmacologic approach tailored to a child’s age and development is fundamental in managing symptoms and chronic pain [5]. The different drugs that can be used to manage chronic pain belong mainly to four categories—non opioids, opioids, adjuvants, and local anesthetics [45]. The possible routes of administration range from oral, venous, and subcutaneous, to transcutaneous, transmucosal, and aerosol. In some situations, epidural or intrathecal administration or other regional anesthesia techniques are required. Non-drug therapies are currently described as physical, behavioral, and cognitive, depending on whether they primarily work by influencing children’s sensory systems, behaviors, or thoughts. Techniques include distraction, attention, imagery, relaxation, and behavioral management [46,47]. Proper drug dosages and the respect of the best practice to manage pain (see Supplementary Table S1) ensure good control of the symptoms in most cases.

Psychological concerns are also widespread in children with cancer, with anxiety, sadness, depression, fear, boredom, and behavioral disorders being the most common symptoms [48]. Spiritual crises are also frequent, especially among older children; on the other hand, hope and faith often represent a protective factor that can sustain resilience and uphold the dignity of life [49]. The identification and management of psychological concerns in children and adolescents with cancer would improve treatment outcomes and the quality of life. Therefore, these findings may guide PPC specialists and families to become more cognizant of these disorders [48].

Social concerns are also frequent in children with cancer, especially in older ones. Social isolation due to frequent hospitalizations is frequently observed, underling the importance of home care [5]. In addition, the shame of social image and a poor social understanding of the disease’s status are commonly reported [50].

Different needs may coexist and influence each other, requiring a comprehensive approach to treat the global suffering of the child. Physical needs represent the main contributor to global suffering, but they are often influenced by psychosocial distress and vice versa [51]. Furthermore, psychological support should be made available during the entire duration of the disease, and a psychologist should be engaged in all critical communication, with the aim of helping the child and family with their ability to cope with the difficult experience and to build resilience to adversity [52].

Remarkably, children’s ability to express their distress differs according to age, cognitive and psychological status, and understanding level of the situation. Older children may suffer more from psychosocial than younger ones, whereas younger children can suffer more from procedures or physical distress.

It is, however, important to remember that children’s needs are often reported by third parties. Indeed, due to age-related or cognitive-related communication issues of children in PPC, caregivers often refer to any need, with an inherent risk of overestimation or underestimation of the actual burden of symptoms [6,53]. In particular, the needs of children with cognitive impairment are often underestimated [54].

Therefore, efforts have been made to validate assessment tools based on child self-reporting symptoms. Different scales for assessment of the burden of symptoms and QoL have been specifically validated in pediatric palliative oncology, such as the PROMIS (Pediatric-Reported Outcome Measurement Information System) form [55,56], PEDsQL scale [57], and the Memorial Symptom Assessment (MSAS) [58]. Their use is recommended to identify the most appropriate scale according to the child’s age, cognitive status, and culture [5].

### 3.2. Family Needs

Pediatric cancer is also a family illness [6]. Indeed, physical, psychosocial, and spiritual concerns extend from the child to the whole family (parents, siblings, and other family members). The needs of family members may vary according to their specific roles in the care of the child, type of relationship with the child, age, cognitive status and understanding level, and cultural and spiritual beliefs [59,60]. Moreover, family engagement in decision-making can be influenced by their cultural background [61]. Reinforcing good parenting, re-establishing behavioral rules and family routines, providing home care wherever possible, and supporting hope (emphasizing positive thoughts, avoiding false hope, and redirecting hope beyond survival) are major parts of the family care of a child with cancer [62]. PPC providers must investigate the family’s actual level of understanding of the illness and prognosis, and adapt communication accordingly [63]. All approaches should consider the specific culture of the family, the family structure, any ongoing conflicts, triggers of emotional distress, and financial issues [6]. Parents must be supported and guided to actively listen and respond to their child’s concerns and feelings without imposing their anxiety or grief.

### 3.3. Communication Needs

Trusting and empathic communication is crucial in both pediatric oncology and palliative care, as it helps promote continuity of care and to address all needs [31]. Moreover, good communication among all participants helps define treatment preferences and goals of care, and promotes the child’s participation in decision making [5]. Clinicians should encourage families to include children and adolescents in decision discussions in an age-appropriate way, and address fears, answer questions, and provide anticipatory guidance [64,65]. Importantly, communication is not a one-time event: doubts or fears should be investigated throughout the disease.

### 3.4. Ethical Needs

Ethical issues are of major importance in PPC. It is widely accepted that the child’s “best interest” should be the goal of care. However, defining the “best interest” is not always easy, especially in complex situations. The final decision is often a resume of multiple options on the basis of the specific clinical situation, with additional attention to the child and family’s cultural values and beliefs [5].

The decision to withhold or withdraw therapy is another area of particular concern; “futility of treatment”, “harming treatment”, and “poor QoL” are defined as ethical reasons for limiting or withholding treatment, but a unique definition of these notions has not been provided yet, and therefore decision making should be evaluated case by case [5]. Bioethics consultation and a multidisciplinary team may be helpful [66].

### 3.5. Team Needs

Most current education curricula, both at the undergraduate and graduate levels, lack time dedicated to PPC, making it difficult to disseminate and improve palliative care in this setting [22,67]. Recent data reported that dedicated educational efforts could improve the decision-making process and communication among healthcare providers and between healthcare providers and families of children in PPC [68,69]. Professionals involved in the PPC setting should receive dedicated comprehensive training on PPC principles (EPEC, curricula, and simulation-based program) [70], coordination of the interdisciplinary team [71], debriefing, and support in difficult decision-making [72]. Effective PPC relies on interprofessional collaboration to achieve shared goals. Therefore, PPC education should also provide opportunities for interprofessional education and clinical training [73,74].

## 4. Models of PPC in Oncology

Several models for integrating palliative care into the management of oncological diseases have been proposed for the adult population, while evidence in the pediatric setting is still poor [75]. Therefore, the definition of strategies for the early integration of PPC in pediatric oncology has been identified as a research priority [76]. However, the application of any model should be reported to the actual availability of resources, with the idea that any physician can universally apply the basic principles of palliative care.

Models that rely on the mere delegation to the oncologist to decide whether to involve palliative care providers are currently discouraged [76]. A possible model relies on the early integration of palliative care principles into pediatric oncology. This model was described by Waldman in the “day-2 talk”, which consists of a second interview ideally following the first diagnostic interview, in which issues of palliative care, such as hope, fears and worries, support, and understanding, are explored [77]. Oncologists can conduct this interview without specific expertise and training in palliative care. A second model comprises the involvement of an oncologist (physician or nurse) with dedicated training in PPC [38,78]. The role of this model is to act as a link between the oncology team, the child and family, and the specialist palliative care team when available. The application of this model was associated with significantly earlier access to PPC and a lower number of hospitalizations in the 90 days before death [79]. However, the limited number of oncologists with specific training in PPC limits the applicability of this model in clinical practice.

While these two models can be considered when a dedicated PPC team is not available, a third model is now considered to be the most widely applicable and the most effective, called the “integrated care model” [80,81,82]. It is based on the synergistic integration of the PPC team with the onco-hematology team from the diagnosis, who routinely refer patients to palliative care for their supportive care needs [80]. Indeed, an interdisciplinary approach implies that all team members are committed, share the same care goal, and mutually collaborate, respecting specific areas of expertise. Moreover, this approach allows patients to rapidly resolve multiple physical and emotional issues, as well as more personalized use of consultants. Some considerations for proper implementation of the “integrated care model” are reported in Table 3.

## 5. End-of-Life Care and Advanced Care Planning

### 5.1. Basic Principles and Symptom Management

There is no unequivocal definition for EoL, and often this period is identified only retrospectively [83]. In palliative care, the term “EoL” usually defines the few days just before death, where it is almost certain and close in time. EoL frequently represents the moment of maximum suffering for the child and family, with a rapid, unpredictable, and challenging evolution. The support of a PPC service improves the outcome of children with cancer at EoL [84,85]. In the EoL period, the main goal is relief from suffering. It is essential to frequently communicate with the child and family to assess the burden of suffering and to coordinate the multidisciplinary team involved in the child’s care in any setting (home, hospice, or hospital). The most common symptoms presented by pediatric patients at the EoL are pain, fatigue, dyspnea, reduced motility, poor appetite, cachexia, nausea and vomiting, weakness, difficulty to swallow, anxiety, sadness and depression, and delirium [86,87]. Symptom assessment and prompt management should be guaranteed continuously. Attention should also be paid to nutrition, skin care, and bowel and urinary function. Bleeding and symptomatic anemia are frequently experienced by children with oncological diseases in the EoL period. The transfusion regime should not be considered aprioristically disproportionate, but rather its application should be evaluated according to symptoms relevance and not only on laboratory findings [88]. Similarly, non-invasive ventilation can be seen as a potential treatment for suffering relief in children with severe dyspnea [83]. However, at EoL, any therapeutic intervention must be balanced between the risks and benefits, and palliative sedation when refractory symptoms are associated with extreme suffering can be proposed [89].

### 5.2. Advanced Care Planning

In a trusting relationship among clinicians, the child, and the family, the hope of cure does not preclude the recognition of incurable diseases, and can gradually move to advanced care planning (ACP). ACP allows patients and clinicians to come together to make decisions, in the context of collaborative communication, considering scientific evidence and the patients’ and family’s values, goals, and preferences. When to start ACP remains an issue as there is value in doing it at any stage of the disease. Parents’ timely planning and preparedness are crucial to avoid difficult conversations during crises and to ensure the coordination of care; therefore, ACP should be initiated well before EoL. Indeed, ACP has a role in defining EoL care by defining the appropriate level of intervention, treatment discontinuation, symptom treatments, and resuscitation. Palliative sedation must be proposed in the presence of symptoms that are intolerable and resistant to any type of therapy/strategy implemented (e.g., in case of pain, dyspnea, and delirium). The aim is, through the reduction of consciousness, for full control of the symptoms.

The most frequently used drugs are benzodiazepines (midazolam in continuous infusion), opioids (fentanyl, morphine for continuous infusion, or other transdermal or subcutaneous opioids), or anesthetics in continuous infusion at low dosage (propofol) [90]. The child’s and parents’ perspectives on setting preferences, spiritual/cultural practices and beliefs, and any “unexpressed wishes” of the child should be investigated, and discussion on after death care should not be avoided [6,91,92].

### 5.3. After Death Care

Death does not represent the end of care. It is essential to treat the body with respect and according to parents’ wishes, culture, and religious practices.

Moreover, after death care should be proposed to the family after the child’s death. Grief after death has a physical, psychological, social, and cultural dimension. It could be perceived differently by family members, and it can be more intense and prolonged when referring to the death of a child [93]. The family should be allowed to have a meeting with the PPC team, including a psychologist, several weeks after the death of the child, to share thoughts on the child’s diseases evolution, EoL care, and family’s doubts or regrets.

## 6. Conclusions and Future Directions

A child with cancer needs care beyond cancer treatment, such as treating complex symptoms, psychosocial support, and sharing existential and spiritual issues. The goal of PPC is to share strategies, tools, and skills with pediatric oncologists to guarantee a continuous and multidimensional assessment and management of those needs, and taking care of the patient−family unit. Early involvement of a PPC team and the application of the PPC principles has been shown to positively impact the QoL of pediatric cancer patients at all stages of the disease.

Unfortunately, there is still a gap between what could be done and what is being done in clinical practice. Indeed, there are several areas to be addressed to allow all children with cancer an integrated and effective approach to palliative care: first, training that allows and stimulates integration between pediatric oncologists and PPC experts, through the acquisition and sharing of skills on the fundamental principles of palliative care and teamwork. Another important need is research: collecting and sharing data, strategies, and tools can lead to new strategies and more effective models of care. To reach all children eligible for PPC and their families, new technologies and tools, such as telemedicine, should be implemented.

Research is also needed to evaluate the effectiveness of therapies and devices for controlling symptoms and suffering, and for developing new effective treatment tools. We also need efforts to define specific approaches in different contexts and to identify shared and measurable indicators of the quality of care. Furthermore, the evaluation of clearer and shared eligibility criteria for PPC of children with oncological diseases allows for defining the extent of needs, the need for specific services, and guide a correct and necessary allocation of resources.

Another area of implementation is social information: it is important that everyone knows the role and objectives of PPC in pediatric oncology and that every parent or child can fearlessly accept them as part of the treatment when proposed.

With the hope that the availability of PPC services will increase worldwide, we believe that it is now essential that the application of palliative care principles becomes familiar to all healthcare professionals. Today in pediatric oncology, holistic care of the child with cancer and their family represents a standard of care that must be guaranteed to every child in every setting.

Moreover, a constantly evolving field, the challenge for the future will be to harmonize the interaction between PPC specialists and oncologists in order to provide truly holistic patient care.

## Figures and Tables

**Table 1:** “Green lights” to consider for the request of specialized PPC for children with cancer.

At diagnosis Life-threatening illness (e.g., extended brain glioma) or advanced-stage cancer (e.g., stage IV neuroblastoma; solid metastatic tumor) Diagnosis of a tumor with an event-free survival rate estimation <40% with current therapies.
During illness Progressive metastatic disease Recurrent or resistant diseases, also after organ failure Major toxicity during treatment In case of prolonged hospitalization (>3 weeks) or prolonged admission to intensive care unit (>1 week) without signs of improvement In case of three or more unplanned hospitalizations for serious medical issues within a 6-month period
Related to complex needs Difficulties in symptoms management, in particular of pain Major psychosocial stress or limited social support Introduction of new devices (gastrostomy or tracheostomy) requiring complex care during the transition from hospital to home Difficulties in decision-making or communication processes

Data from [30,31].

**Table 2:** Indications according to the different stages of disease and after death.

	Diagnosis	Progression	End of Life	After Death Care
Symptom management	Early screening and institution of therapy for symptom control for the child and family, as appropriate Engage both the child and parents in symptom reporting	Provide frequent reassessment of symptoms Offer 24-h specialized assistance in case of severe symptoms Ensure management of symptoms appropriate with age and developmental stage	Provide 24/7 assistance (in the presence or from remote) Ensure the continuity of care (home–hospice–hospital)	
Management of psychological, social, spiritual needs	Early screening for psychological, social, and existential distress in the child Offer psychological support, in particular to manage grief and the feeling of loss Support and help the child in maintaining peer relationships and attending school Reinforcement of parenting	Provide frequent reassessment Offer psychological support Discuss wishes and preferred setting of life Discuss wishes and preferred modality of after death care	Provide specialized psychological and spiritual support for the child and family Make the family and child a part of the care plan Facilitate connections with bereaved peers	Facilitate connections with bereaved peers Help connection with formal and informal support in the community
Assessment of the quality of life	Support and help family restore their daily routine	Limit futile interventions Guide the family through the advanced care planning Investigate child and family idea of QoL and share strategies for its achievement	Balance any intervention on risk and benefit Act with the aim to guarantee comfort	
Communication	Communicate clearly and honestly with child and family for a trusting relationship Verify the correct understanding of illness and prognosis Encourage the sharing of private feelings, in particular those related to bereavement	Provide a clear and honest discussion with the child and family about prognosis, not limiting hope Engage the child in the decision-making process Encourage the sharing of private feelings, in particular those related to bereavement	Provide clear and honest communication about the EoL evolution Define the EoL setting according to the wishes of the child and family	Provide a clear and honest review of the child history to bring out emotions and feelings
Family support	Assess the family needs Evaluate the presence of a supportive network for the family	Provide frequent reassessment of the family needs	Assess the family needs during the EoL	Provide support during after-death care Help family dealing with grief Offer psychological and spiritual support to parents, siblings, and other family members Allow bereaved family members the opportunity to reconnect with the PPC team to be affirmed their child’s life is honored and remembered
Coordination activities	Support oncologist in introducing PPC principle since the diagnosis Support oncologists in difficult clinical or ethical scenarios	PPC team and oncologists share the responsibilities of difficult decision-making and communication Ensure the respect of the child’s preferences	Coordinate the health care network in all settings so as to respect the child’s preferences Offer in-hospice or home-based specialized assistance	Coordinate the health care network in assisting the family during after-death care Offer debriefing support to the health care providers involved in the child care

**Table 3:** Considerations for PPC providers and oncologists to properly implement the “integrated care model”.

Prognosis is difficult to define due to a lack of standardized criteria for defining incurability and the rapid increase of new technologies or therapeutic innovations. The patient could experience a rapid and unpredictable evolution to the terminal stage, with a high risk of catastrophic symptoms (i.e., bleeding, sepsis). The patient may need frequent access to a hospital, even as death approaches, for blood tests and imaging. Referral to PPC should be based on needs rather than life expectancy. The PPC team can offer advice and symptom management without conflicting with the treatment goals. PC can be offered alongside oncological therapies, including involvement in clinical trials. Establishing a close and trusting relationship between the patient and PPC team is as important as establishing a relationship between the patient and the oncologist.

## Supplementary Materials

The following supporting information can be downloaded at https://www.mdpi.com/article/10.3390/cancers14081972/s1. Table S1: Best practices to manage pain in children with cancer.
