# Supplementary material for: Pediatric Palliative Care in Oncology: Basic Principles
Source: Cancers (Basel). 2022 Apr 13;14(8):1972. doi: 10.3390/cancers14081972 (PMC9031296; doi:10.3390/cancers14081972)
Supplement: Supplementary file 1 [file cancers-14-01972-s001.zip › cancers-1634498-supplementary.pdf]

Pediatric palliative care in oncology: basic principles

Table S1. Best practices to manage pain in children with cancer.

**Multimodal approach**	Provide a combination of pharmacological and non-pharmacological therapy, with the use of synergistic drugs. The advantages of this practice are, in addition to the integrated action on the causes and perceptive mechanisms of pain, the opportunity to reduce side effects and enhance analgesia.
**Diagnosis-targeted therapy**	Provide a therapy based on the clinical and etiopathogenetic diagnosis of pain and on the "situation target", according to the extent of the pain, the expected duration of therapy and the patient's condition (age, clinical and emotional situation).
**Respectful management**	Provide fixed-time administration, through the choice of the simplest, most effective and least painful route of administration; pay attention to the circadian rhythm, provide additional doses of medication in case of acute pain, prophylaxis of predictable pain.
**Sharing of therapeutic choices**	Share with patient and family the analgesic program, providing information and promoting involvement and empowerment
**Continuous monitoring**	Provide pain monitoring with validated scales, appropriate to age and situation, regarding the effectiveness and occurrence of possible side effects
